# The association between electronic-cigarette use and self-reported oral symptoms including cracked or broken teeth and tongue and/or inside-cheek pain among adolescents: A cross-sectional study

**DOI:** 10.1371/journal.pone.0180506

**Published:** 2017-07-11

**Authors:** Jun Ho Cho

**Affiliations:** Department of Public Health Administration, Hanyang Women’s University, Seoul, Republic of Korea; National Yang-Ming University, TAIWAN

## Abstract

**Background:**

Little is known about oral health related to electronic-cigarette (EC) use, even though EC use is increasing rapidly. The aim of this study is to assess the relationship between EC use and oral health, including ‘gingival pain and/or bleeding’, ‘tongue and/or inside-cheek pain’, and ‘cracked or broken teeth’ among adolescents.

**Methods:**

A total of 65,528 students in 2016 were included in this cross-sectional study.

**Results:**

For EC use, 0.5% (n = 297) students were daily users, 1.9% (n = 1259) were ‘1 to 29 days past month users’, and 5.9% (n = 3848) were former users. Overall, 18.5% students reported they had experienced ‘gingival pain and/or bleeding’, 11.0% reported ‘tongue and/or inside-cheek pain’, and 11.4% reported a ‘cracked or broken tooth’ within the past 12 months. When comparing ‘daily EC users’, ‘1 to 29 days past month EC users’, and ‘former EC users’ with ‘never EC users’, the adjusted ORs for ‘cracked or broken tooth’ were 1.65 (95% CI: 1.19–2.27), 1.26 (95% CI: 1.06–1.51), and 1.16 (95% CI: 1.04–1.30), respectively. Comparing ‘daily EC users’ with ‘never EC users’, the adjusted OR for ‘tongue and/or inside-cheek pain’ was 1.54 (1.05–2.26). However, EC use among adolescents was not associated with ‘gingival pain and/or bleeding’ when adjusted for the potential confounders.

**Conclusions:**

Based on the results, the odds of cracked or broken teeth among daily, ‘1 to 29 days past month’, and former EC users were significantly higher than those among never EC users. The odds of tongue and/or inside-cheek pain among daily EC users were significantly higher than those among never EC users. In conclusion, the results suggest that daily EC use among adolescents may be a risk factor for cracked or broken teeth and tongue and/or inside-cheek pain.

## Introduction

Electronic cigarettes (EC) are battery-powered electronic devices, which aerosolize liquid that contains nicotine, humectants, and flavors [1]. EC use has increased rapidly and globally, particularly among smokers and adolescents [2]. During 2010–2013, ever EC use increased among current conventional cigarettes (CC) smokers (9.8%–36.5%) and among former CC smokers (2.5%–9.6%) in a study of US adults. Among Korean adolescents, ever EC use was 0.5% in 2008 and increased to 8.2% in 2014 [3]. In Poland, ever EC use among high school students increased from 16.8% in 2010/11 (n = 1,760) to 62.1% in 2013/14 (n = 1,970) [4]. The North Carolina Youth Tobacco Survey found that prevalence of use in the past 30 days increased from 1.7% in 2011 (n = 4,791) to 7.7% in 2013 (n = 4,092) [5]. The issues regarding their effectiveness as a smoking cessation aid and health risks due to EC use are still controversial [6]. So far, there is no strong evidence in regards to their safety, although there are reports that ECs may be less harmful to users and bystanders, than CCs [7]. It is known that the main reasons for using ECs are to quit CCs, as an alternative to CCs, curiosity, appealing flavors, and peer influences [8, 9].

Oral disease is one of the most common public health issues worldwide and constitutes a significant socio-economic burden [10]. Oral health is an important part of the quality of life among adolescents [11] and can influence school attendance [12]. Over 7% of American children have already lost at least one tooth in their lifetime because of cavities by the age of 17 [13]. Biology, lifestyle, and environment are important factors of oral health [14]. Tobacco products are one of the risk factors for oral health. For example, CC smoke impairs innate defenses against pathogens, modulates antigen presentation and immunity in the oral cavity, and promotes gingival and periodontal disease and oral cancer [15]. Additionally, the messenger RNA expression of dentin matrix acidic phosphoprotein-1, bone sialoprotein, and alkaline phosphatase activity significantly decreased in nicotine-treated human dental pulp cells, and mineralized nodule formation was also inhibited [16]. Namely, nicotine inhibits the cytodifferentiation and mineralization of human dental pulp cells, possibly via nicotinic acetylcholine receptors. Besides, a recent experimental research reported that EC aerosols caused cytotoxicity to oral epithelial cells, and the molecular mechanisms might be due to oxidative stress induced by toxic substances present in EC aerosols [17]. Moreover, ECs increased inflammatory and pro-senescence responses in oral epithelial cells and periodontal fibroblasts [18]. A previous report to dental professionals has recommended that all patients should be advised about the unknown dangers of ECs because there were no product standards that would control levels of dosing, chemicals, or carcinogens in the solution used in ECs or the aerosols [19].

Even though EC use is increasing rapidly, little is known about oral health related to EC use. There has never been a representative population study assessing the association of EC use with oral health among adolescents or among adults. Therefore, the aim of this study was to assess the association between EC use and oral symptoms that includes ‘gingival pain and/or bleeding’, ‘tongue and/or inside-cheek pain’, and ‘cracked or broken teeth’ among adolescents in South Korea.

## Methods

### Study population

The Twelfth Korean Youth Risk Behavior Web-based Survey (KYRBWS) was approved by an institutional review board of the Korean Center for disease Control and Prevention (2014-06EXP-02-P-A). This study was reviewed by the Institutional Review Board of Hanyang Women’s University and complied with ethical requirements (AN01-201504-HR-010-01). Data used was from the Twelfth KYRBWS, 2016, Ministry of Education, Ministry of Health and Welfare, and Korean Center for Disease Control and Prevention [3, 20]. The understanding, reliability and validity of the questions were investigated by the Centers for Disease Control and Prevention of Korea (KCDC) [21]. The Eleventh KYRBWS provides a representative sample of all middle and high school students in Korea, ranging from 7^th^ to 12^th^ school grade students. The population was sampled from 400 middle and 400 high schools. Out of 67,983 students, 65,528 students responded, an overall response rate of 96.4% from 798 schools. Out of 33,251 middle school students, 32,219 students responded, an overall response rate of 96.9%. Out of 34,732 high school students, 33,309 students responded, an overall response rate of 95.9%.

### Outcome definition

Oral symptoms were defined as an outcome on a student’s self-report. Students were asked the question: “Within the past 12 months, have you experienced gingival pain and/or bleeding?” (yes/no). Students were also asked the question: “Within the past 12 months, have you experienced tongue and/or inside-cheek pain?” (yes/no). Students were lastly asked the question: “Within the past 12 months, have you experienced a cracked or broken tooth?” (yes/no).

### EC use

EC use was defined by the question, “Have you ever used an EC in your life, even one or two puffs?” (yes/no). A no answer was categorized as ‘never user.’ Respondents who answered in the positive were asked the next question: “During the past 30 days, on how many days have you used ECs?” Respondents answering ‘none’ were categorized as a ‘former user.’ Positive responses were re-categorized into two groups: ‘1 to 29 days past month user: 1 to 29 days use’ and ‘daily user: all 30 days use.’ A report of the Surgeon General on smoking assessed current CC smoking prevalence for youth and young adults based on having smoked all or part of at least one cigarette in the past 30 days [22]. Similarly, current EC users are usually defined as adolescents who indicated use in the past 30 days. In this study, however, in order to assess the daily EC use effects, we re-classified the ‘past 30 day users’ into two groups as above. First EC experience was defined by the question: “When did you experience ECs for the first time?” Response options were re-categorized into five groups: ‘never EC users,’ ‘10^th^– 12^th^ grade’, ‘7^th^ -9^th^ grade’, ‘1^st^ - 6^th^ grade’, and ‘<1^st^ grade.’ We also assessed the reasons for using ECs applying the questions “What is the main reason for using ECs?” The response options were ‘it seems to be healthier than CCs’, ‘to quit smoking CCs’, ‘to use them indoors’, ‘it is easier to get ECs than CCs’, ‘good taste’, ‘good flavors’, ‘doesn’t smell bad’, ‘curiosity’, and ‘other.’ We assessed sources from which EC users acquire EC-liquids using the questions “How do you usually get EC-liquids?” The response options were ‘from friends’, ‘purchase from an EC shop’, ‘purchase through the internet’, ‘other’, and ‘only purchase nicotine free EC-liquids.’ The response options were also re-classified into two groups: ‘nicotine-free EC user in the past 30 days’ and ‘nicotine-containing EC user in the past 30 days.’ Unless explicitly specified, all EC fluids in Korea contain nicotine due to their popularity. Also, only ECs which contain nicotine have been classified as tobacco products by the Ministry of Finance (MOF) since 2010 in Korea [23]. Length of time in years from first experience with EC was calculated by subtracting ‘first EC experience grade’ from ‘current school grade’.

### Socio-demographic and other variables

Age, gender, school grade, city size, economic status, stress, overweight status, carbonated drinks, alcohol, vigorous sports activity, CC smoking, attempt to quit CC smoking, and second hand smoke at home were assessed. The questions were as follows: For economic status, “What is your household’s economic status?” The response options were ‘very high’, ‘high’, ‘middle’, ‘low’, and ‘very low.’ Stress was also investigated (S1 Table), as a statistically significant association between perceived current stress levels and dental insurance on oral pain has been found [24]. The response options for the question regarding stress, “Usually, how often do you feel stress?” were classified into 5 groups: ‘Never’, ‘rarely’, ‘sometimes’, ‘most of the time’, and ‘always.’ The questions for carbonated drinks and alcohol consumption were, “During the past 7 days, how often did you drink carbonated drinks?” and “During the past 30 days, on how many days did you drink alcohol, even a glass of alcohol?” The response options were ‘none’, ‘1–2 days’, ‘3–5 days’, ‘6–9 days’, ‘10–19 days’, ‘20–29 days’, and ‘daily.’ The question for physical activity was, “During the past 7 days, on how many days were you physically and vigorously active for a total of at least 20 minutes per day (※vigorous activities: jogging, soccer, basketball, mountain climbing, rapid biking, rapid swimming, taekwondo *etc*.).” Overweight status was calculated using the body mass index (BMI: calculated as weight in kilograms divided by height in meters squared). Overweight status was classified into two groups: ‘BMI ≥ 25 kg/m^2’^ and ‘BMI < 25 kg/m^2^.^’^ CC smoking was assessed by the question, “Have you ever smoked a CC in your life, even one or two puffs?” (yes/no). A no answer was classified as ‘never smoker.’ Respondents who answered in the affirmative were asked a follow-up question: “During the past 30 days, on how many days have you smoked CCs?” Respondents answering ‘none’ were classified as a ‘former smoker.’ Affirmative responses were re-classified into two groups: ‘1 to 29 days past month smoker: 1 to 29 days use’ and ‘daily smoker: all 30 days use.’ Attempt to quit smoking was assessed by the question: “During the past 12 months, have you ever attempted to quit CC smoking?” (yes/no). A yes answer was classified as ‘attempt to quit smoking.’ Second hand smoking at home was assessed by the question: “During the past week, on how many days were you exposed to second hand smoke at home?” All response options are shown in detail in Table 1.

**Table 1:** Characteristics of the study population composed of adolescents in 2016.

Characteristics		No. (65528)	% (100)
Mean age (y) ± SD		14.99 ±1.74(SD)	
Gender	Male	33803	51.6
	Female	31725	48.4
School	7^th^ grade	10483	16.0
	8^th^ grade	10517	16.0
	9^th^ grade	11219	17.1
	10^th^ grade	11355	17.3
	11^th^ grade	11070	16.9
	12^th^ grade	10884	16.6
Economic status	Very high	6247	9.5
	High	17997	27.5
	Middle	31056	47.4
	Low	8324	12.7
	Very low	1904	2.9
City type	Large	33666	51.4
	Middle/small	27885	42.6
	Rural	3977	6.1
Stress	Never	2389	3.6
	Rarely	10772	16.4
	Sometimes	28021	42.8
	Most of the time	17833	27.2
	always	6513	9.9
Overweight status	BMI < 25	55610	84.9
	BMI ≥25	8131	12.4
	Missing	1787	2.7
Carbonated drinks	None per week	15895	24.3
	1–2 per week	31893	48.7
	3–4 per week	12325	18.8
	5–6 per week	2819	4.3
	Once per day	1354	2.1
	Twice per day	622	.9
	Thrice per day	620	.9
Alcohol	None in the past 30 days	15375	23.5
	1–2 days in the past 30 days	5719	8.7
	3–5 days in the past 30 days	1818	2.8
	6–9 days in the past 30 days	866	1.3
	10–19 days in the past 30 days	587	.9
	20–29 days in the past 30 days	267	.4
	daily	172	.3
	Never drink	40724	62.1
Vigorous sports activity	None per week	15034	22.9
	1 time per week	12785	19.5
	2 times per week	12429	19.0
	3 times per week	10067	15.4
	4 times per week	4796	7.3
	≥5 times per week	10417	15.9
Oral health outcome			
Gingival pain and/or bleeding	Yes	12096	18.5
	No	53432	81.5
Tongue or inside-cheek pain	Yes	7237	11.0
	No	58291	89.0
Cracked and/or broken tooth	Yes	7500	11.4
	No	58028	88.6
Tobacco			
Conventional cigarette (CC) use	Never CC smoker	56017	85.5
	Former smoker	5499	8.4
	1 to 29 days past month	2109	3.2
	Daily smoker: all 30 days	1903	2.9
Attempt to quit CC smoking	Yes	2824	4.3(70.4)^a^
	No	1188	1.8(29.6)^a^
	NA	61516	93.9
Second hand smoking at home	None per week	46023	70.2
	1 time per week	5099	7.8
	2 times per week	4378	6.7
	3 times per week	3144	4.8
	4 times per week	1515	2.3
	5 times per week	1131	1.7
	6 times per week	579	.9
	Daily	3659	5.6
Electronic cigarette (EC) use	Never EC user	60124	91.8
	Former user	3848	5.9
	1 to 29 days past month	1259	1.9
	Daily user: all 30 days	297	.5
First EC experience	Never EC user	60124	91.8
	≥10^th^ grade	1708	2.6(31.9)^b^
	7^st^-9^th^ grade	3093	4.7(57.7) ^b^
	1^st^-6^th^ grade	401	.6(7.5) ^b^
	<1^st^ grade	159	.2(2.9) ^b^

^a^; The percentages in parenthesis are for past 30 day CC smokers.

^b^; The percentages in parenthesis are for ever EC users.

### Statistical analysis

The data were described by descriptive statistics and analyzed using binary-logistic regression analyses (IBM SPSS Version 23.0). First, frequency analyses were conducted to assess the rate of oral health outcome and EC use, including sociodemographic variables. Second, to assess the unadjusted odds ratios (ORs) indicating association between EC use and oral health outcome, we applied binary-logistic regression analyses. Third, to assess the adjusted ORs, we applied multiple logistic regression analyses including the potential confounders; age, gender, school grade, economic status, and city size. Third, to assess the final adjusted ORs, we performed multiple logistic regression analyses including the above in addition to carbonated drink, overweight status, stress, alcohol, vigorous sports activity, CC smoking, attempt to quit smoking, and second hand smoking at home. Logical errors (for example, a male reported in a female only school) and outlier values (for example, age, height, weight, BMI), are treated as missing values according to the statistical analysis guidelines of the KYRBWS. The proportion of respondents treated as missing value was below 2%. Missing data were handled by using pairwise deletion.

## Results

### Population characteristics

The frequencies of the adolescent characteristics are shown in Table 1. The mean age for the students was 15.0 (SD: 1.7). A total of 51.6% were male. Overall, 18.5% students reported that they had experienced ‘gingival pain and/or bleeding’ within the past 12 months. Approximately, 11.0% students reported that they had experienced ‘tongue and/or inside-cheek pain’. In addition, 11.4% students reported that they had experienced a ‘cracked or broken tooth.’ Overall, 9.9% felt stress always, and 12.4% were overweight. About 3.9% drank carbonated drinks every day and 0.3% had at least one drink of alcohol every day. 15.9% were physically and vigorously active on more than 5 days per week. For CC smoking, 2.9% students were daily CC smokers, 3.2% were ‘1 to 29 days past month CC smokers’, and 8.4% were former CC smokers. Among daily and ‘1 to 29 days past month’ CC smokers, 70.4% students had tried to quit smoking during the past 12 months. In addition, 5.6% students were exposed to second hand smoke at home every day.

**Fig 1:**
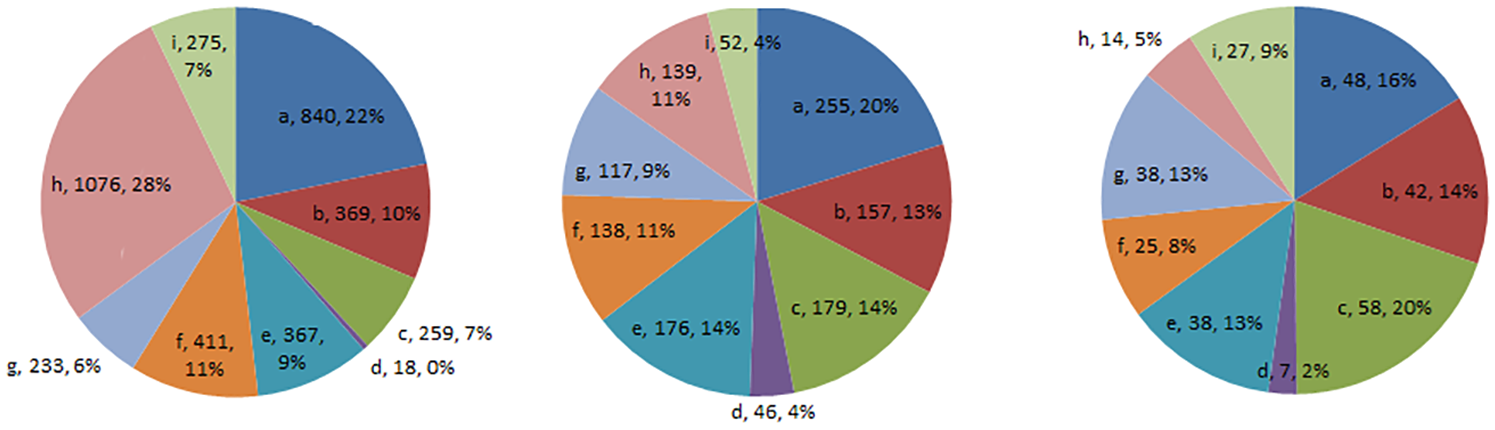
Main reason for using ECs among adolescents. **Fig 1.1. Former EC users. Fig 1.2. 1 to 29 days past month EC users. Fig 1.3. Daily EC users**. a) It seems to be healthier than CCs, b) To quit smoking CCs, c) To use it indoors, d) It is easier to get ECs than CCs, e) Good taste, f) Good flavors, g) Doesn’t smell bad, h) Curiosity, i) Other.

For EC use, 0.5% (n = 297) of students were daily EC users, 1.9% (n = 1259) were ‘1 to 29 days past month EC users’, and 5.9% (n = 3848) were former EC users during the past month. About 91.8% (n = 60124) were never EC users. For first EC experience time, amongst ever EC users, 2.9% (n = 159) had experienced ECs before 1^st^ grade and 7.5% (n = 401) had experienced ECs between 1^st^ and 6^th^ grade. With relation to the main reason for EC use, ‘to use ECs indoors’ was the most common reason among daily EC users (Fig 1).

As a reason, ‘curiosity’ tapered off as frequency of use increased. ‘Curiosity’ accounted for 28% of former EC users, 11% among ‘1 to 29 days past month EC users’, and 5% among daily EC users.

**Fig 2:**
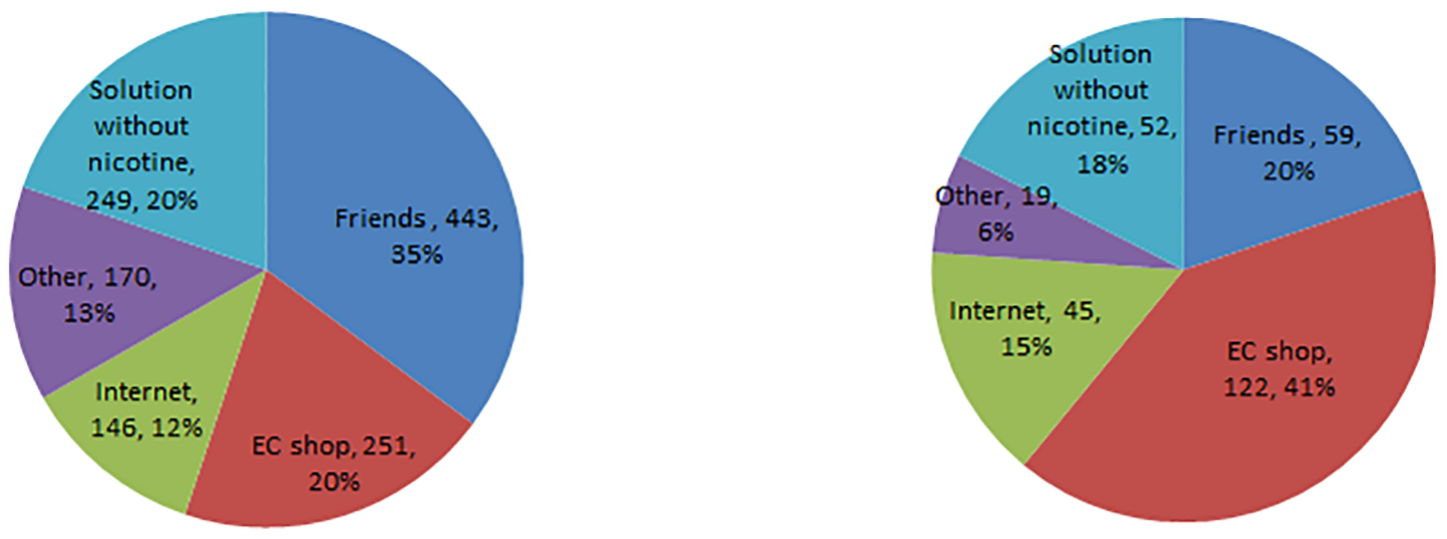
How to get EC-liquids among adolescent EC users. **Fig 2.1. 1 to 29 days past month EC users. Fig 2.2. Daily EC users**. Of 1259 ‘1 to 29 days past month’ EC users, 20% (n = 249) students bought nicotine-free EC solutions and 80% (n = 1010) students bought nicotine-containing EC solutions. Of 297 daily EC users, 18% (n = 52) students bought nicotine-free EC solutions and 82% (n = 245) students bought nicotine-containing EC solutions.

Among ‘1 to 29 days past month EC users’, 35% (n = 443) reported friends as a primary source (Fig 2). Among daily EC users, 41% (n = 122) reported EC shops as a primary source.

Table 2 shows the length of time from first experience with EC among ‘1 to 29 days past month’ and daily EC users by school grade.

**Table 2:** Length of time from first experience with EC among ‘1 to 29 days past month’ and ‘daily EC’ users by school grade.

Length of time from first experience with EC among ‘1 to 29 days past month’ EC users (year)	% by school grade						Total (n = 1248)
	7^th^ (n = 34)	8^th^ (n = 87)	9^th^ (n = 157)	10^th^ (n = 273)	11^th^ (n = 343)	12^th^ (n = 354)	
<1	58.8	46.0	28.0	16.8	12.0	12.7	18.9
1	29.4	41.4	33.8	33.3	29.4	23.4	30.0
2	8.8	3.4	17.8	25.6	26.2	32.8	24.8
3		1.1	1.9	7.0	14.0	7.9	7.9
4			1.3	1.5	3.5	5.4	3.0
5	2.9		1.9	.4	.6	3.7	1.6
6		2.3	1.3	.7	1.5		.9
7		3.4	3.2	1.5	1.5	.8	1.6
8		2.3	4.5	5.5	1.5	.8	2.6
9			6.4	5.1	3.8	2.5	3.7
10				2.6	4.7	2.8	2.6
11					1.5	5.6	2.0
≥12						1.4	.4
Total	100.0	100.0	100.0	100.0	100.0	100.0	100.0
Length of time from first experience with EC among daily EC users (year)	% by school grade						Total (n = 291)
	7^th^ (n = 7)	8^th^ (n = 18)	9^th^ (n = 39)	10^th^ (n = 49)	11^th^ (n = 54)	12^th^ (n = 124)	
<1		16.7	7.7	16.3	7.4	9.7	10.3
1		33.3	20.5	20.4	27.8	26.6	24.7
2		5.6	17.9	24.5	18.5	28.2	22.3
3		5.6	10.3	2.0	9.3	7.3	6.9
4	28.6			2.0		4.8	3.1
5		5.6				4.0	2.1
6						.8	.3
7	71.4	16.7	2.6	4.1		.8	4.1
8		16.7	2.6		3.7	.8	2.4
9			38.5	2.0	1.9	.8	6.2
10				28.6		1.6	5.5
11					31.5	.8	6.2
≥12						13.7	5.8
Total	100.0	100.0	100.0	100.0	100.0	100.0	100.0

The number of ‘1 to 29 days past month’ and daily EC users increased by school grade. Furthermore, amongst daily and ‘1 to 29 days past month’, most students had their first EC experience ‘equal to or more than one year ago’. Among ‘1 to 29 days past month’ EC users, 18.9% had their first experience ‘less than one year ago’ and 81.9% ‘equal to or more than one year ago’. Among daily EC users, 10.3% had their first EC experience ‘less than one year ago’ and 89.7% ‘equal to or more than one year ago’.

Table 3 shows the numbers of students by frequency of use of ECs and CCs.

**Table 3:** Cross tab showing the dual uses between EC use and CC smoking.

		EC user (%)				
		Never	Former	1 to 29 days past month	Daily	Total (%)
CC smoker	Never	55126 (98.4)	655 (1.2)	196 (0.3)	40 (0.1)	56017 (100.0)
	Former	3662 (66.6)	1697 (30.9)	118 (2.1)	22 (0.4)	5499 (100.0)
	1 to 29 days past month	925 (43.9)	657 (31.2)	479 (22.7)	48 (2.3)	2109 (100.0)
	Daily	411 (21.6)	839 (44.1)	466 (24.5)	187 (9.8)	1903 (100.0)
	Total	60124 (91.8)	3848 (5.9)	1259 (1.9)	297 (0.5)	65528 (100.0)

Of daily CC smokers (n = 1903), 9.8% (n = 187) students were daily EC users. Of ‘1 to 29 days past month’ CC smokers (n = 2109), 2.3% (n = 48) students were daily EC users.

### Association of CC smoking with oral health

The ORs for CC smoking were used for a preliminary comparison. The adjusted ORs indicating association between CC smoking and oral symptoms among adolescents are shown in Table 4.

**Table 4:** Multivariable adjusted odds ratios showing association between CC smoking and oral symptoms among adolescents.

CC smoking	Oral symptoms			
	No.	Gingival pain and/or bleeding: Adjusted OR (95% CI)	Tongue and/or inside-cheek pain: Adjusted OR (95% CI)	Cracked or broken tooth: Adjusted OR (95% CI)
Never smoker	56017	1	1	1
Former smoker	5499	1.10 (1.02–1.19)*	1.16 (1.05–1.28)**	1.25 (1.14–1.37)***
1 to 29 days past month smoker	2109	1.14 (0.95–1.35)	1.02 (0.82–1.28)	1.13 (0.93–1.38)
Daily smoker	1903	0.98 (0.81–1.18)	0.80 (0.62–1.02)	1.33 (1.08–1.63)**

Adjusted OR; adjusted for the age, gender, school grade, economic status, and city size, carbonated drink, overweight status, stress, alcohol, vigorous sports activity, EC use, attempt to quit smoking, and second hand smoking at home

*; p < 0.05,

**; p < 0.01,

***; p < 0.001.

For ‘cracked and/or broken tooth,’ comparing ‘daily CC smoker’ with ‘never CC smoker’, the adjusted OR was 1.33 (1.08–1.63). For ‘cracked and/or broken tooth,’ comparing ‘former CC smoker’ with ‘never CC smoker’, the adjusted OR was 1.25 (1.14–1.37). Other oral health measures were statistically insignificant or showed statistically significant differences with ORs close to 1.0.

### Association of EC use with ‘gingival pain and/or bleeding’

The ORs indicating association between EC use and ‘gingival pain and/or bleeding’ among adolescents are shown in Table 5.

**Table 5:** Multivariable odds ratios showing association between EC use and ‘gingival pain and/or bleeding’ among adolescents.

EC use	Gingival pain and/or bleeding				
	No (%)	Yes (%)	Model 1 OR (95% CI)	Model 2 OR (95% CI)	Model 3 OR (95% CI)
Never user	49160 (81.8)	10964 (18.2)	1	1	1
Former user	3062 (79.6)	786 (20.4)	1.15 (1.06–1.25)**	1.22 (1.12–1.33)***	0.98 (0.88–1.08)
1 to 29 days past month user	985 (78.2)	274 (21.8)	1.25 (1.09–1.43)**	1.25 (1.08–1.44)**	0.88 (0.74–1.05)
Daily user	225 (75.8)	72 (24.2)	1.44 (1.10–1.87)**	1.41 (1.05–1.90)*	1.00 (0.72–1.41)

Model 1; unadjusted

Model 2; adjusted for age, gender, school grade, economic status, and city size

Model 3; adjusted for age, gender, school grade, economic status, city size, carbonated drink, overweight status, stress, alcohol, vigorous sports activity, CC smoking, attempt to quit smoking, and second hand smoking at home

*; p < 0.05,

**; p < 0.01,

***; p < 0.001

Comparing ‘daily EC users’ with ‘never EC users’, the adjusted OR was 1.41 (95% CI: 1.05–1.90) in Model 2 and 1.00 (95% CI: 0.72–1.41) in Model 3. Good fits to the data according to the Hosmer-Lemeshow χ^2^-test were produced in Model 2 (χ^2^ = 8.0, df = 8, p = 0.432) and in Model 3 (χ^2^ = 7.4, df = 8, p = 0.492).

### EC use increased odds of ‘tongue and/or inside-cheek pain’

The ORs indicating association between EC use and ‘tongue and/or inside-cheek pain’ among adolescents are shown in Table 6.

**Table 6:** Multivariable odds ratios showing association between EC use and ‘tongue and/or inside-cheek pain’ among adolescents.

EC use	Tongue and/or inside-cheek pain				
	No (%)	Yes (%)	Model 1 OR (95% CI)	Model 2 OR (95% CI)	Model 3 OR (95% CI)
Never user	53549 (89.1)	6575 (10.9)	1	1	1
Former user	3417 (88.8)	431 (11.2)	1.03 (0.93–1.14)	1.14 (1.02–1.27)*	0.98 (0.86–1.11)
1 to 29 days past month user	1087 (86.3)	172 (13.7)	1.29 (1.10–1.52)**	1.31 (1.10–1.56)**	1.08 (0.88–1.33)
Daily user	238 (80.1)	59 (19.9)	2.02 (1.52–2.69)***	2.04 (1.48–2.81)***	1.54 (1.05–2.26)*

Model 1; unadjusted

Model 2; adjusted for age, gender, school grade, economic status, and city size

Model 3; adjusted for age, gender, school grade, economic status, city size, carbonated drink, overweight status, stress, alcohol, vigorous sports activity, CC smoking, attempt to quit smoking, and second hand smoking at home

*; p < 0.05,

**; p < 0.01,

***; p < 0.001

Comparing ‘daily EC users’ with ‘never EC users’, the adjusted OR was 2.04 (95% CI: 1.48–2.81) in Model 2 and 1.54 (95% CI: 1.05–2.26) in Model 3. Namely, when adjusting for the potential confounders, the odds of ‘tongue and/or inside-cheek pain’ among daily EC users was over 50% higher (p = 0.028) than that among never EC users. Further, good fits to the data according to the Hosmer-Lemeshow χ^2^-test were produced in Model 2 (χ^2^ = 7.5, df = 8, p = 0.481) and in Model 3 (χ^2^ = 10.3, df = 8, p = 0.248).

### EC use increased odds of ‘cracked or broken tooth’

The ORs indicating association between EC use and ‘cracked or broken tooth’ among adolescents are shown in Table 7.

**Table 7:** Multivariable odds ratios showing association between EC use and ‘cracked or broken tooth’ among adolescents.

EC use	Cracked or broken tooth				
	No (%)	Yes (%)	Model 1 OR (95% CI)	Model 2 OR (95% CI)	Model 3 OR (95% CI)
Never user	53605 (89.2)	6519 (10.8)	1	1	1
Former user	3202 (83.2)	646 (16.8)	1.66 (1.52–1.81)***	1.62 (1.48–1.77)***	1.16 (1.04–1.30)*
1 to 29 days past month user	1005 (79.8)	254 (20.2)	2.08 (1.81–2.39)***	1.93 (1.67–2.24)***	1.26 (1.06–1.51)*
Daily user	216 (72.7)	81 (27.3)	3.08 (2.39–3.99)***	2.87 (2.16–3.82)***	1.65 (1.19–2.27)**

Model 1; unadjusted

Model 2; adjusted for age, gender, school grade, economic status, and city size

Model 3; adjusted for age, gender, school grade, economic status, city size, carbonated drink, overweight status, stress, alcohol, vigorous sports activity, CC smoking, attempt to quit smoking, and second hand smoking at home

*; p < 0.05,

**; p < 0.01,

***; p < 0.001

Comparing ‘daily EC users’ with ‘never EC users’, the adjusted OR was 2.87 (95% CI: 2.16–3.82) in Model 2 and 1.65 (95% CI: 1.19–2.27) in Model 3. Finally, in Model 3, the odds of ‘cracked or broken tooth’ among daily EC users was over 60% higher (p = 0.003) than that among never EC users. In addition, the odds of ‘cracked or broken tooth’ among former EC users (OR 1.16, CI 1.04–1.30) and ‘1 to 29 days past month’ EC users (OR 1.26, CI 1.06–1.51) were significantly higher than those among never EC users. Furthermore, good fits to the data according to the Hosmer-Lemeshow χ^2^-test were produced in Model 2 (χ^2^ = 9.3, df = 8, p = 0.316) and in Model 3 (χ^2^ = 14.4, df = 8, p = 0.071). Just for supporting information, using the weighted no. and the weighted %, we assessed multivariable adjusted odds ratios showing association between EC use and oral symptoms among adolescents (S2 Table). The weighted results were similar to the unweighted results.

### Association of nicotine-free or nicotine-containing EC use with oral symptoms

The adjusted ORs indicating association between nicotine-free or nicotine-containing EC use and oral symptoms among adolescents are shown in Table 8.

**Table 8:** Multivariable adjusted odds ratios showing association between EC use according to nicotine content and oral symptoms among adolescents.

EC use	Oral symptoms			
	No.	Gingival pain and/or bleeding: Adjusted OR (95% CI)	Tongue and/or inside-cheek pain: Adjusted OR (95% CI)	Cracked or broken tooth: Adjusted OR (95% CI)
Never EC user	58680	1	1	1
Former EC user	3716	0.98 (0.89–1.09)	0.97 (0.86–1.11)	1.16 (1.04–1.30)*
Nicotine-free EC user in the past 30 days	212	0.83 (0.58–1.18)	1.56 (1.07–2.28)*	1.12 (0.78–1.61)
Nicotine-containing EC user in the past 30 days	1133	0.91 (0.76–1.08)	1.07 (0.86–1.32)	1.37 (1.15–1.63)**

Adjusted OR; adjusted for age, gender, school grade, economic status, city size, carbonated drink, overweight status, stress, alcohol, vigorous sports activity, CC smoking, attempt to quit smoking, and second hand smoking at home

Missing; n = 1787.

*; p < 0.05,

**; p < 0.01.

For ‘cracked and/or broken tooth,’ the adjusted OR was 1.37 (1.15–1.63) when comparing ‘nicotine-containing EC users in the past 30 days’ with ‘never EC users.’ According to the Hosmer-Lemeshow χ^2^-test for this analysis the model provided a good fit to the data (χ^2^ = 13.6, df = 8, p = 0.093). For ‘tongue and/or inside-cheek pain,’ the adjusted OR was 1.56 (1.07–2.28) when comparing ‘nicotine-free EC users in the past 30 days’ with ‘never EC users.’ According to the Hosmer-Lemeshow χ^2^-test for this analysis the model provided a good fit to the data (χ^2^ = 8.7, df = 8, p = 0.368). For ‘gingival pain and/or bleeding,’ there were no significant associations between EC use according to nicotine content and oral symptoms. Therefore, of the studied oral conditions, ‘cracked and/or broken tooth’ was significantly associated with the use of nicotine-containing ECs, and ‘tongue and/or inside-cheek pain’ was significantly associated with the use of nicotine-free ECs.

## Discussion

This study evaluated the association between EC use and oral symptoms among adolescents. It found that EC use was associated with a significantly increased chance of either cracked or broken teeth or tongue and/or inside-cheek pain among adolescents. However, the results did not find an association between EC use and gingival pain and/or bleeding among adolescents.

Nicotine may be a contributing factor in cases of cracked or broken teeth. This hypothesis is supported by the significant association between nicotine-containing EC users and cracked or broken teeth. This hypothesis is also supported by the report that the messenger RNA expression of dentin matrix acidic phosphoprotein-1, bone sialoprotein, and alkaline phosphatase activity were significantly decreased in nicotine-treated human dental pulp cells of smokers, and mineralized nodule formation was also inhibited by nicotine in human dental pulp cells [16]. Namely, the functions of dentin matrix synthesis and mineralization may be decreased in the dental pulp cells of smokers. This hypothesis is also upheld by the recent findings that direct exposure of human dental pulp cells to nicotine results in an inflammatory response that could have a role in pulpal inflammation onset, a pathological condition that may eventually progress to pulp necrosis [25]. Dental caries can have serious and lasting complications. A recent report demonstrated a positive association between dental trauma and dental caries in permanent teeth [26]. Nicotine also enhances *Streptococcus mutans* biofilm formation and biofilm metabolic activity, increasing the development of caries [27]. Acid production of biofilms decreases the local pH to a level that boosts demineralization of the dentin and enamel, resulting in potential synergism between chemical and mechanical modes [28]. EC inhalation has been shown to alter innate immunity and increase the virulence of colonizing bacteria [29]. In addition, a previous study reported that EC could not deliver nicotine to the blood stream at levels equal to tobacco cigarettes within the same time-period of use, and suggested that nicotine from EC aerosols are not absorbed from the lungs but from the oral mucosa, that nicotine absorption occurs at a similar rate to nicotine-replacement therapies, and that a significant part of the nicotine deposited to the oral mucosa seemed to be swallowed [30]. Xerostomia, due to EC use, may be related to cracked or broken teeth [31]. Xerostomia causes a reduction in the crack growth resistance of dentin [32]. In general, xerostomia may be related to a reduction in the level of salivary flow rate due to aging, some medications or other conditions [33]. The hypothesis is supported by the review that the common health hazards of EC use include dry mouth, mouth or tongue sores/inflammation, and dryness of the mucus membrane [34]. Lead, due to solder from EC, may be one of the causes for cracked or broken teeth. This hypothesis is supported by the report that environmental lead exposure was associated with an increased prevalence of dental caries among children aged 5 to 17 years in the US population [35]. In addition, lead was measured in the leachate of disposed electronic cigarettes at levels as high as 50mg/L by the Waste Extraction Test and 40mg/L by the Toxicity Characteristic Leaching Procedure [36].

Regardless of nicotine, the thermal degradation byproducts formed from glycerin, propylene glycol, and flavorings during vapor generation may be a cause of tongue and/or inside-cheek pain. This hypothesis is supported by the additional result of this study that tongue and/or inside-cheek pain was significantly associated with the use of nicotine-free ECs. Also, this hypothesis is endorsed by an in-vitro experimental study, conducted to explore the effects of ECs on the oropharynx that, independently of nicotine, ECs induced DNA strand breaks and cell death [37]. Aldehydes including acrolein caused an increase in cellular oxidative stress in a keratinocytic model of oral exposure, probably due to the reduction in glutathione (GSH) levels [38]. Acetaldehyde, acrolein, and formaldehyde from ECs, especially the higher levels of aldehydes from the newer-generation EC devices, indicated the risks of using ECs [39]. Acrolein was primarily produced by glycerin degradation, and acetol and 2-propen-1-ol were produced mostly from propylene glycol, while formaldehyde originated from both [40]. Also, e-liquids with flavorings showed particularly high ranges of chemicals, causing concerns about their potential toxicity in case of chronic oral exposure [41]. In addition, thermal decomposition of flavoring compounds from e-liquids dominated toxic aldehyde production during EC vaping [42]. According to a previous study, among US students who had ever used electronic vaporizer such as an EC, 65–66% students used electronic vaporizers in order to vaporize ‘just flavoring’ in 12th, in 10th and in 8th grade, and to vaporize other substances including marijuana (about 5–7%) [43]. Thus, those who use ECs without nicotine for just flavorings are likely to be exposed to the flavoring compounds more than those who use ECs with nicotine containing fluids. In a previous study, ever and ‘1 to 29 days past month’ EC users had higher odds of reporting that flavored ECs were less harmful than non-flavored ECs, compared to youths who did not use ECs [44]. Further, the average puff duration of the nicotine-free liquids was significantly longer compared to the nicotine-containing liquids (36 mg/ml) [45]. Also, liquid consumption and puff number were higher for the low nicotine strength liquids [46]. Nickel, from the nickel-chromium heating filaments of ECs, may be one of the causes for tongue and/or inside-cheek pain. This hypothesis is endorsed by earlier studies that nickel in metal crowns induced genotoxicity in buccal epithelial cells in children [47]. And a previous study of 22,083 patients who were diagnosed with oral cancer between 1982 and 2002, which suggested nickel might be a new risk factor for oral cancer [48]. This is also supported by a study that found nickel in farm soils is probably a common environmental risk factor for esophageal cancer and oral cancer [49]. Nickel was found to be 2–100 times higher in concentration in EC aerosol than in Marlboro brand cigarettes [1]. However, other substances not yet studied in e-liquids may be a cause of ‘tongue and/or inside-cheek pain’ as drug inhalation using EC devices is a new trend [50].

Conversely, we were not able to identify the association between EC use and ‘gingival pain and/or bleeding’ among adolescents in this study. Our results for ‘gingival pain and/or bleeding’ do not coincide with the research that ECs with flavorings cause increased oxidative/carbonyl stress and inflammatory cytokine release in human periodontal ligament fibroblasts [51]. Besides, it is still questionable why electronic cigarettes only affect the tongue and/or inside-cheek, even though the gums are also soft tissues. Therefore, further research is required to assess the risk of using ECs on periodontal disorders. However, it is possible that the vaping behavior of EC users compared to CC smokers is one of the reasons, especially regarding puffing topography. This is supported by the study that found ECs required stronger vacuums to vape than CCs, and necessitated increased puff strength to produce an aerosol [52]. Also, ECs were smoked more intensively than CCs because of the puffing difference between EC users and CC smokers [53]. Therefore, to produce stronger vacuums and draw in more aerosols, EC users might have to use their tongues, palates, and inside-cheeks together. This may expose e-vapor or e-liquid directly to the tongue and inside-cheek rather than the gums. This is supported by the fact that liquid leakage is a common complaint of EC users [54].

Given the cross-sectional study design, these findings need to be interpreted with caution. The results cannot establish a causal relationship between EC use and oral health. Furthermore, due to a reliance on self-reported oral symptoms without oral examination, we should admit recall bias. Thus, dental surgeon’s medical records for oral health including cracked or broken teeth and tongue and/or inside-cheek pain need to be evaluated to confirm the association between EC use and oral health. Also, diet, oral hygiene status, oral hygiene regimen and overjet of teeth were not taken into consideration in this study, even though diet and oral hygiene could be confounding factors in causing caries, broken teeth, and bleeding or pain. In addition, the strength of EC use, for example nicotine dose per day of EC use, was not considered. As some EC users started because they perceived it as less harmful or helpful when quitting CCs. These health-oriented EC users may have experienced complications (e.g. poor oral health) as a result of smoking CCs, before trying ECs. At the same time, those users who only smoke CCs but not ECs may have been less susceptible to complications from CC use. Another limitation is the difference in referenced timeframes between ‘EC use during the past 30 days’ and ‘oral health within the last 12 months’, which do not exactly represent current health status. However, this study is supplemented by detailed information on EC users, such as exposure time from first experience with EC and the main reason for EC use by type of EC user. The numbers of EC users in the study, in particular, daily users, were relatively small. The statistical reliability of the study would have been better if the sample size of EC users were larger. In future EC research, the main cause of oral health problems needs to be identified by oral examination. Despite the potential limitations, it is notable that this is the first large representative population study to evaluate the association of EC use with oral health, using daily EC use and recent data from 2016.

## Conclusions

Based on the results, after adjusting for potential confounders, EC use was associated with a significantly increased chance of either cracked or broken teeth, or tongue and/or inside-cheek pain among adolescents. The odds of cracked or broken teeth among daily, ‘1 to 29 days past month’, and former EC users were significantly higher than those among never EC users. The odds of tongue and/or inside-cheek pain among daily EC users were significantly higher than those among never EC users. In conclusion, the results suggest that EC use among adolescents may be a risk factor for tongue and/or inside-cheek pain and cracked or broken teeth.

## Supporting information

S1 Table Adjusted odds ratios showing the stress effects on oral symptoms among adolescents. (PDF)

S2 Table Multivariable adjusted odds ratios showing the association between EC use and oral symptoms among adolescents, using weighted no. and weighted %. (PDF)
