# Supplementary material for: The association between electronic-cigarette use and self-reported oral symptoms including cracked or broken teeth and tongue and/or inside-cheek pain among adolescents: A cross-sectional study
Source: PLoS One. 2017 Jul 11;12(7):e0180506. doi: 10.1371/journal.pone.0180506 (PMC5507461; doi:10.1371/journal.pone.0180506)
Supplement: S1 Table — (PDF) [file pone.0180506.s001.pdf]

S1 Table. Adjusted odds ratios showing the stress effects on oral symptoms among adolescents

Stress	No.	Oral symptoms		
		Gingival pain and/or bleeding: Adjusted OR (95% CI)	Tongue and/or inside-cheek pain: Adjusted OR (95% CI)	Cracked and/or broken tooth: Adjusted OR (95% CI)
Never	2389	1	1	1
Rarely	10772	1.38 (1.19-1.61)***	1.37 (1.13-1.67)**	0.85 (0.74-0.98)*
Sometimes	28021	2.02 (1.75-2.32)***	1.93 (1.60-2.32)***	0.98 (0.86-1.12)
Most of the time	17833	2.88 (2.49-3.32)***	2.98 (2.47-3.59)***	1.10 (0.96-1.26)
Always	6513	3.77 (3.25-4.38)***	4.22 (3.49-5.11)***	1.30 (1.13-1.51)***

Adjusted OR; adjusted for EC use.

*; $p < 0.05$, **; $p < 0.01$, ***; $p < 0.001$

All three models produced a good fit to the data according to the Hosmer-Lemeshow χ^2-test (data not shown).
