# Supplementary material for: The association between electronic-cigarette use and self-reported oral symptoms including cracked or broken teeth and tongue and/or inside-cheek pain among adolescents: A cross-sectional study
Source: PLoS One. 2017 Jul 11;12(7):e0180506. doi: 10.1371/journal.pone.0180506 (PMC5507461; doi:10.1371/journal.pone.0180506)
Supplement: S2 Table — (PDF) [file pone.0180506.s002.pdf]

S2 Table. Multivariable adjusted odds ratios showing the association between EC use and oral symptoms among adolescents, using weighted no. and weighted %.

EC use	Weighted no.	Weighted %	Oral symptoms		
			Gingival pain and/or bleeding: Adjusted OR (95% CI)	Tongue and/or inside-cheek pain: Adjusted OR (95% CI)	Cracked and/or broken tooth: Adjusted OR (95% CI)
Never user	2844492	91.7	1	1	1
Former user	185333	6.0	0.98 (0.89-1.09)	1.02 (0.90-1.15)	1.15 (1.03-1.28)*
1 to 29 days past month	57842	1.9	0.87 (0.73-1.04)	1.06 (0.85-1.32)	1.29 (1.09-1.52)**
Daily user	12027	0.4	0.97 (0.68-1.38)	1.50 (1.03-2.19)*	2.00 (1.44-2.77)***

Adjusted OR; adjusted for the age, gender, school grade, economic status, and city size, carbonated drink, overweight status, stress, alcohol, vigorous sports activity, CC smoking, attempt to quit smoking and second hand smoking at home.

Missing; n = 1787.

*; $p < 0.05$, **; $p < 0.01$, ***; $p < 0.001$
